# Intermittent screening and treatment with dihydroartemisinin-piperaquine and intermittent preventive therapy with sulfadoxine-pyrimethamine have similar effects on malaria antibody in pregnant Malawian women

**DOI:** 10.1038/s41598-019-44340-x

**Published:** 2019-05-27

**Authors:** Andrew Teo, Louise M. Randall, Mwayiwawo Madanitsa, Victor Mwapasa, Linda Kalilani Phiri, Carole Khairallah, Christelle Buffet, Amalia Karahalios, David L. Narum, Feiko O. Ter Kuile, Stephen J. Rogerson

**Affiliations:** 1 Department of Medicine and Radiology and Doherty Institute, University of Melbourne, Melbourne, Victoria Australia; 2 Laboratory of Malaria Immunology and Vaccinology, National Institute of Allergy and Infectious Diseases, National Institute of Health, Rockville, Maryland USA; 3 Victoria Infectious Diseases Service, Peter Doherty Institute of Infection and Immunity, University of Melbourne, Melbourne, Victoria, Australia; 4 College of Medicine, University of Malawi, Blantyre, Malawi; 5 Department of Clinical Sciences, Liverpool School of Tropical Medicine, Liverpool, United Kingdom; 6 Centre of Epidemiology and Biostatistics, Melbourne School of Population and Global Health, The University of Melbourne, Melbourne, Victoria Australia; 7 Liverpool School of Tropical Medicine, Liverpool, UK

**Keywords:** Malaria, Malaria, Epidemiology

## Abstract

In a randomised trial comparing intermittent screening and treatment (IST) with dihydroartemisinin-piperaquine (DP) and intermittent preventive therapy against malaria in pregnancy (IPT) with sulfadoxine-pyrimethamine (SP) in Malawi, the impacts of IST-DP and IPT-SP on the development and maintenance of malaria antibody immunity were compared. Pregnant Malawian women were randomised to receive IST-DP or IPT-SP. In a nested study, paired enrolment and delivery plasma samples from 681 women were assayed for antibodies against recombinant antigens and for IgG and opsonising antibodies to antigens found on infected erythrocytes (IEs). At delivery, antibody responses did not differ between study arms. Between enrolment and delivery, antibodies to recombinant antigens decreased, whereas antibodies to IEs including opsonising antibodies remained stable. Overall, changes in antibody responses over pregnancy did not differ by treatment arm. Stratifying by gravidity, antibody to schizont extract decreased more in multigravidae receiving IST-DP than IPT-SP. There was minimal impact of treatment arm on the development and maintenance of malaria immunity. While antibodies to recombinant antigens declined between enrolment and delivery, antibodies directed against IEs tended to be more stable, suggesting longer-lasting protection.

Clinical trial registration: Pa n African Clinical Trials Registry (PACTR201103000280319) 14/03/2011. URL: http://www.isrctn.com/ISRCTN69800930.

## Introduction

Malaria in pregnancy (MiP) increases the risk of mortality and morbidity in pregnant women and their infants^1^. A first-time pregnant mother is at the highest risk, but intermittent preventive therapy during pregnancy (IPT) can reduce the impact of MiP. Sulfadoxine-pyrimethamine (SP) is the only drug recommended for IPT, but emergence of resistance threatens ongoing efficacy^2,3^.

One alternative strategy for malaria prevention is intermittent screening and treatment (IST), in which a rapid diagnostic test (RDT) is performed at each scheduled antenatal visit, and if the RDT is positive, participants are given effective antimalarial drug(s).

In pregnancy, *Plasmodium falciparum* infected erythrocytes (IEs) sequester in the placenta, and these IEs express VAR2CSA, a unique variant surface antigen (VSA) that binds to the placental receptor chondroitin sulfate A (CSA)^4^. Antibody targeting placental-binding IEs is acquired over successive pregnancies, and is associated with protection against MiP and its consequences^5,6^. Most studies of antibody responses have used samples collected during the last trimester or at delivery, but pregnant women begin to acquire antibody to placental-binding IEs early in first pregnancy^7,8^, and the use of IPT-SP has been shown to slow acquisition of such immunity^9^. IST relies on RDT-based detection of infection and will not detect placental-sequestered parasites or submicroscopic infections, thus potentially exposing pregnant women to longer periods of parasitaemia than IPT. Whether this affects the development of pregnancy-specific immunity, or the maintenance of malaria immunity more generally, is unknown, but studies mainly in non-pregnant hosts show that antibody is important in clearance of malaria infection, including infections with drug-resistant parasites^10,11^.

Using samples from 681 pregnant Malawian women participating in a clinical trial of IPT-SP compared to IST with dihydroartemisinin-piperaquine (DP)^12^, we evaluated the impact of IST or IPT on acquisition and maintenance of malarial immunity. We compared antibody responses at study enrolment and delivery and change in antibody responses from enrolment to delivery by treatment arm, and evaluated the effect of malaria infection during pregnancy on antibody measurements.

## Results

### Participants’ characteristics

At enrolment, participants’ characteristics were similar between women receiving IPT-SP (N = 333) and IST-DP (N = 348), except that women receiving IPT-SP were slightly heavier (mean; standard deviation = 54.9 kg; 7.3) than those receiving IST-DP (53.0 kg; 6.8), Table 1. During follow-up, more women in the IPT-SP arm experienced febrile episodes (8.2% vs 1.1%, for IPT-SP and IST-DP, respectively), and at delivery, there were fewer LBW deliveries in the IPT-SP arm (10.5% vs 15.7% for IPT-SP and IST-DP, respectively; Table 1).

**Table 1:** Study population characteristics at enrolment and delivery in IPT-SP and IST-DP arms.

At enrolment	Intermittent preventive treatment (SP) (N = 333)	Intermittent screening and treatment (DP) (N = 348)
**Study site**		
Madziabango	103 (30.9)	117 (33.6)
Mpemba	110 (33.0)	112 (32.2)
Chikwawa	120 (36.0)	119 (34.2)
Age, years	20 (18–23)	20 (18–22)
**Gravidity**		
Gravida 1	165 (49.5)	161 (41.9)
Gravida 2	117 (35.1)	118 (33.9)
Gravida 3+	51 (15.3)	68 (19.5)
Weight, kg	54.7 (7.3)	53.7 (6.8)
Height, cm (SD)	154.1 (4.9)	153.5 (4.7)
BMI, kg/m^2^ (SD)	24.0 (2.9)	22.8 (2.8)
Haemoglobin, g/dl (SD)	10.7 (1.4)	10.7 (1.4)
Anaemia at enrolment, Hb < 11.0 g/dl	185 (55.6)	184 (52.9)
Parasitemia (light microscopy)	63/329 (19.1)	62/341 (17.9)
Parasitemia (qPCR)	147/327 (45.0)	177/343 (51.6)
Bed net use at enrolment	68 (20.4)	73 (21.0)
IRS last 12 months	40/329 (12.2)	37/346 (10.7)
**Socio-economic status**		
Low	108 (32.5)	101 (29.1)
Medium	120 (35.8)	134 (38.3)
High	105 (31.6)	112 (32.6)
**At delivery**		
Haemoglobin, g/dL (SD)	11.7 (1.5)	11.8 (1.6)
Anaemia at delivery	98/326 (30.0)	84/336 (25.0)
Placental malaria on histology ^b^Infected^b^, n/N (%)	109/300 (36.3)	122/324 (37.6)
Peripheral parasitemia^a a^(qPCR)	60/317 (18.9)	79/337 (23.4)
Newborn sex male,	181/326 (55.6)	166/336 (49.4)
Birth weight, g (SD)	2897 (447)	2863 (406)
Low birth weight <2500 g (%)	34/323 (10.5)	52/332 (15.7)

NOTE. Data presented as n (%) or mean (SD), unless otherwise indicated. DP, dihydroartemisinin-piperaquine; SP, sulfadoxine-pyrimethamine; SD, standard deviation; BMI, body mass index; Hb, haemoglobin; qPCR, quantitative polymerase chain reaction; IRS, indoor residual spraying. ^a^Parasitemia based on microscopy and qPCR. ^b^Infected placentas classified based on histology reading of active or chronic infection.

### Antibody responses to *P*. *falciparum* antigens at delivery

**Figure 1:**
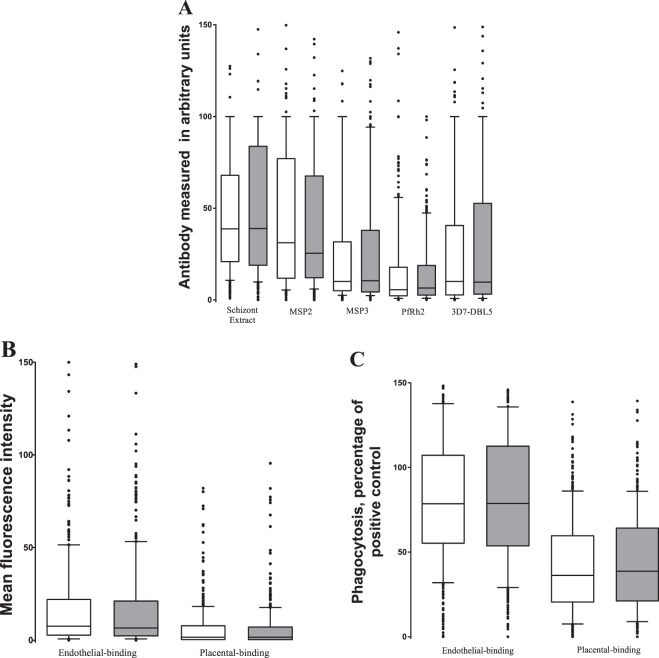
Immunoglobulin G (IgG) antibodies to *P. falciparum* antigens at delivery according to treatment group. White box – Pregnant women receiving IPT-SP, N = 333, grey box – Pregnant women receiving IST-DP, N = 348. (**A**) Levels of IgG to schizont extract and recombinant proteins MSP2, MSP3, PfRH2 and 3D7-DBL5. (**B**) IgG responses to variant surface antigens of endothelial-binding and placental-binding infected erythrocytes (IEs). (**C**) Levels of opsonising IgG to variant surface antigens of endothelial-binding and placental-binding IEs, presented as percentage of THP-1 cells that have ingested IEs (Phagocytosis, relative to positive controls). Data presented in box and whiskers plot box showing median and IQR, and whiskers 10–90 percentiles with outliers in closed circles. Results are presented as percentage of positive controls.

Median antibody responses to schizont extract, recombinant merozoite antigens and median total antibodies or opsonising IgG to endothelial-binding and placental-binding IEs did not differ by treatment arm at delivery (Table 2 and Fig. 1). However, other factors were associated with some of the antibodies measured. Multigravidae had higher antibody responses to several pregnancy-specific antigens than women in first or second pregnancy [3D7-DBL5: coeff = 1.4 (95% CI 1.1, 1.7), P = 0.002; IgG to placental-binding CS2 IEs, coeff = 1.3 (1.1, 1.5), P = 0.01; opsonising IgG to CS2 IEs, coeff = 1.4 (1.3, 1.5), P < 0.0001]. *P*. *falciparum* infection at enrolment was associated with slightly higher IgG responses to MSP3 [coeff = 1.2 (1.0, 1.5), P = 0.04], and opsonising IgG to endothelial-binding IEs [coeff = 1.1 (1.0, 1.2), P = 0.03], but associated with lower total IgG responses to placental-binding IEs [coeff = 0.7 (0.5, 1.0), P = 0.02]. Malarial antibodies also differed by study site. Compared to women from Madziabango, women from Mpemba had lower IgG responses to 3D7-DBL5 [coeff = 0.5 (0.3, 0.9), P = 0.02], and in Chikwawa, they had lower IgG responses to PfRh2, coeff = 0.2 (0.2, 0.4), P < 0.0001; MSP3, coeff = 0.6 (0.5,0.9), P = 0.03; 3D7-DBL5 coeff = 0.2 (0.1, 0.4), P < 0.0001; and opsonising antibody to endothelial-binding IEs coeff = 0.8 (0.7, 1.0), P = 0.04, consistent with the higher parasite prevalence reported in Madziabango in the parent trial^12^. Importantly, there was no evidence of an interaction between intervention arm and study site in their effects on malarial antibodies.

**Table 2:** Linear regression models of antibodies at delivery (analysed as natural log, and antilogged back to antibody units in table), adjusting for confounders and interaction terms.

Variables	IgG schizont extract		IgG PfRh2		IgG MSP2		IgG MSP3		IgG 3D7-DBL5		Total IgG to endothelial-binding IE		Opsonising Ab to endothelial-binding IE		Total IgG to placental-binding IE		Opsonising Ab to placental-binding IE	
	Coeff (95%Cl)	P	Coeff (95%Cl)	p	Coeff (95%Cl)	p	Coeff (95%Cl)	p	Coeff (95%Cl)	p	Coeff (95%Cl)	p	Coeff (95%Cl)	p	Coeff (95%Cl)	p	Coeff (95%Cl)	p
IPT-SP	Ref		Ref		Ref		Ref		Ref		Ref		Ref		Ref		Ref	
IST-DP	1.1 (0.8–1.5)	0.5	1.1 (0.8–1.7)	0.5	0.9 (0.7–1.2)	0.5	1.1 (0.8–1.6)	0.5	1.0 (−0.6–1.7)	0.9	0.9 (0.6–1.4)	0.7	1.0 (0.8–1.1)	0.7	1.1 (0.7–1.7)	0.6	1.1 (0.9–1.4)	0.3
**Gravidity**																		
Paucigravid	Ref		Ref		Ref		Ref		Ref		Ref		Ref		Ref		Ref	
Multigravid	0.9 (0.9–1.1)	0.5	1.1 (0.9–1.3)	0.2	1.1 (0.9–1.2)	0.4	1.1 (1.0–1.3)	0.1	1.4 (1.1–1.7)	0.002	1.1 (1.0–1.4)	0.2	1.0 (0.9–1.1)	0.9	1.3 (1.1–1.5)	0.01	1.4 (1.3–1.5)	<0.0001
***P***. ***falciparum*** **at enrolment**^**a**^																		
No	Ref		Ref		Ref		Ref		Ref		Ref		Ref		Ref		Ref	
Yes	1.1 (1.0–1.3)	0.08	1.2 (0.9–1.5)	0.1	1.1 (0.9–1.3)	0.4	1.2 (1.0–1.5)	0.04	1.1 (0.8–1.4)	0.5	0.8 (0.6–1.1)	0.2	1.1 (1.0–1.2)	0.03	0.7 (0.5–1.0)	0.02	1.0 (0.9–1.2)	0.5
**Study-site**																		
Madziabango	Ref		Ref		Ref		Ref		Ref		Ref		Ref		Ref		Ref	
Mpemba	1.0 (0.7–1.4)	1.0	1.0 (0.6–1.6)	0.9	0.7 (0.5–1.0)	0.08	1.0 (0.7–1.6)	0.8	0.5 (0.3–0.9)	0.02	1.4 (0.9–2.3)	0.1	1.0 (0.8–1.2)	0.8	0.7 (0.4–1.1)	0.1	0.8 (0.7–1.1)	0.1
Chikwawa	0.9 (0.7–1.2)	0.7	0.2 (0.2–0.4)	<0.0001	0.9 (0.6–1.2)	0.3	0.6 (0.5–0.9)	0.03	0.2 (0.1–0.4)	<0.0001	1.4 (0.9–2.2)	0.1	0.8 (0.7–1.0)	0.04	0.8 (0.5–1.3)	0.5	0.5 (0.6–1.5)	0.1
**IST-DP** ^**b**^ **Study sites**																		
Madziabango	Ref		Ref		Ref		Ref		Ref		Ref		Ref		Ref		Ref	
Mpemba	0.7 (0.5–1.1)	0.1	0.8 (0.4–1.3)	0.3	0.9 (0.6–1.4)	0.7	0.8 (0.5–1.3)	0.4	0.9 (0.5–1.8)	0.8	1.1 (0.6–2.0)	0.7	1.0 (0.9–1.3)	0.8	0.8 (0.4–1.7)		1.1 (0.8–1.5)	0.5
Chikwawa	0.9 (0.6–1.3)	0.7	0.9 (0.5–1.5)	0.7	1.0 (0.7–1.5)	0.9	1.2 (0.5–1.4)	0.5	1.0 (0.5–1.8)	0.9	1.3 (0.7–2.3)	0.5	1.1 (0.9–1.3)	0.6	0.7 (0.4–1.4)		0.8 (0.6–1.1)	0.2

Results presented as ratio of geometric means and 95% confidence interval, and P-values. A coefficient between 0–1 implies a decrease of antibody levels. ^a^Infection based on blood smears and quantitative polymerase chain reaction; ^b^IST-DP was fitted into an interaction model with study-sites.

### Changes in antibody responses to *P*. *falciparum* antigens during pregnancy

Median antibody responses to recombinant antigens and schizont extract declined over the course of pregnancy in both treatment arms (Fig. S1), but there were no differences in responses between treatment arms at either time point.

When we examined antibody to IEs, there was an increase in total IgG antibody to endothelial-binding IEs in the IPT-SP arm at delivery, but this association was absent for placental-binding IEs and in the IST-DP arm to both parasite strains, Fig. S1B. The median opsonising IgG responses did not vary between enrolment and delivery in either arm, Fig. S1C. There were no other differences in the antibody responses to IEs VSA at either time point.

### Changes in individual antibody responses during pregnancy between treatment groups

**Figure 2:**
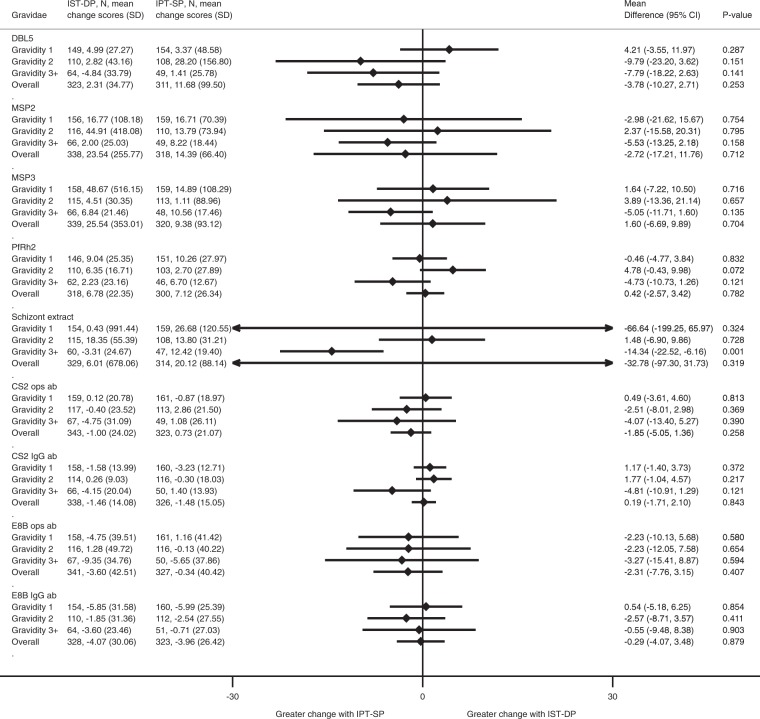
Immunoglobulin G (IgG) antibodies to *P. falciparum* antigens at delivery according to treatment group. Pregnant women receiving IPT-SP, N = 333, Pregnant women receiving IST-DP, N = 348. Mean difference compares the change in antibody response from enrolment to delivery between the IST-DP and IPT-SP treatment arms after adjusting for antibody response at enrolment.

Antibody responses in the IST-DP group varied more between enrolment and delivery than those in the IPT-SP group. In particular, in multigravid women antibody responses to schizont extract increased substantially over pregnancy, [mean = 14.3 (6.2, 22.5) P < 0.001] (Fig. 2).

### Modification of changes in antibody responses from enrolment to delivery by infection

**Figure 3:**
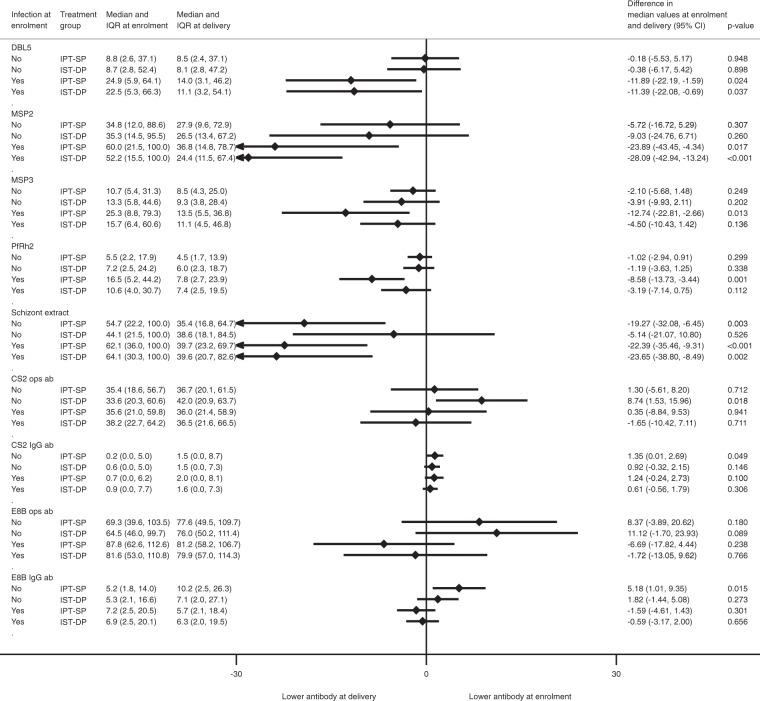
Change in immunoglobulin G (IgG) antibodies to *P. falciparum* antigens from enrolment to delivery stratified by infection status at enrolment and by treatment arm. Median difference compares the change in antibody response from enrolment to delivery between the IPT-SP and IST-DP treatment arms.

Women were stratified according to presence or absence of *P*. *falciparum* at enrolment (by microscopy and/or PCR) in each treatment group. The most striking changes in antibody from enrolment to delivery were seen in women infected at enrolment, and for responses to recombinant antigens and schizont extract, Fig. 3. Antibody to DBL5, MSP2 and schizont extract declined in both treatment arms, while antibody to MSP3 and PfRh2 declined only in the IPT-SP arm. In the IPT-SP group, there were modest increases in antibody to VSA of both CS2 and E8B IEs among uninfected women, and in the IST-DP group, there was an increase in opsonising IgG antibody to CS2 IEs. In contrast, antibody to schizont extract decreased from enrolment to delivery in both groups regardless of infection status at enrolment. Other responses did not change, Fig. 3.

**Figure 4:**
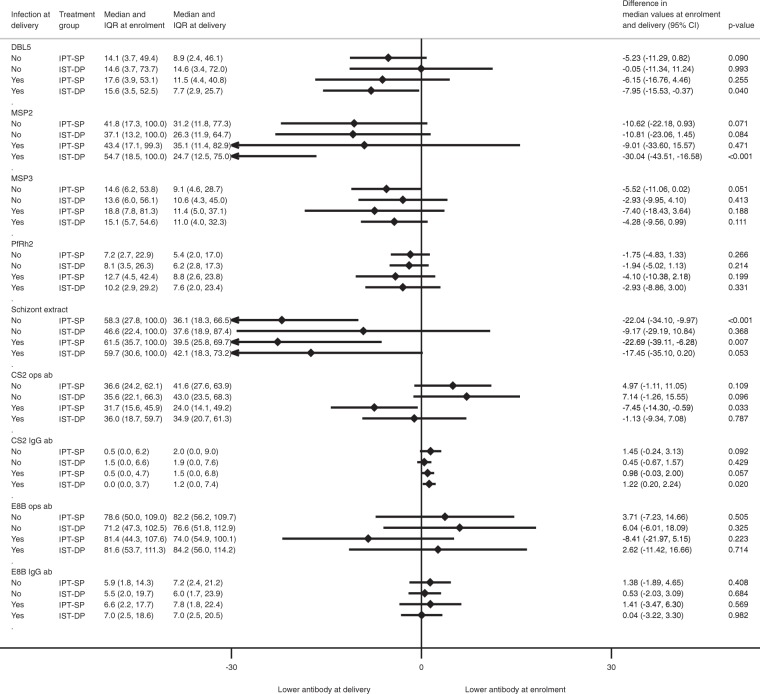
Change in immunoglobulin G (IgG) antibodies to *P. falciparum* antigens from enrolment to delivery stratified by infection status at delivery and by treatment arm. Median difference compares the change in antibody response from enrolment to delivery between the IPT-SP and IST-DP treatment arms.

When stratified based on infection status at delivery (by histology and/or microscopy), infected women in the IST-DP group tended to have increases in antibody to VSA of CS2 IEs, Fig. 4. Interestingly, among women with malaria at delivery, opsonising IgG to CS2 IE and antibody to schizont extract declined from enrolment to delivery in women who received IPT-SP, and among women receiving IST-DP, there were declines in antibody responses to DBL5 and MSP2 from enrolment to delivery, Fig. 4.

## Discussion

In the context of a randomised trial of IST-DP compared to IPT-SP in pregnant Malawian women, we investigated the effect of each treatment on antibody immunity to malaria, and determined whether IST-DP altered the development and maintenance of pregnancy-specific antibody immunity when compared to IPT-SP. Antibodies to recombinant antigens, but not functional antibodies, declined during pregnancy in both arms. There was no evidence of a differential impact on malarial humoral immunity between treatment arms, but when participants were stratified by gravidity, multigravid women receiving IST-DP had increases in antibody to schizont extract from enrolment to delivery, possibly due to increased exposure to low-density malaria infections that were not detected by RDT during scheduled antenatal visits.

At delivery, there were no differences in levels of antibody to schizont extract and recombinant antigens or to antigens expressed on IEs between treatment arms. The lack of broad differences between groups may in part reflect the relatively low incidence of malaria infections and short duration of follow up in this well-protected group of women.

In the absence of effective malaria prevention, pregnant women develop increasing levels of pregnancy-specific immunity between antenatal booking and delivery, with the greatest increases seen in first pregnancies^7,13^. By contrast, in the present study antibodies to schizont extract, merozoite antigens and to VAR2CSA-DBL5 declined over the course of pregnancy, and total IgGs and opsonising antibodies to VSA of placental-binding and endothelial-binding IEs were generally stable, largely consistent with recent studies in Papua New Guinea, Benin and Malawi^5,14,15^. The exception to this was an increase in total IgG responses to endothelial-binding IEs in the IPT-SP group, which may indicate that placental-binding isolates are not the only variants infecting pregnant women^16^. The lack of change in antibody to IEs suggests that haemodilution associated with pregnancy does not explain the changes in antibody responses to recombinant merozoite antigens, VAR2CSA-DBL5 or schizont extract. Instead, their half-lives may be shorter than those for antibodies to VSA^17^. Our findings, together with previous studies, highlight the importance of studying functional antibodies to malaria^18^, potentially including measures of antibody avidity and binding-inhibition antibodies against placental-binding IEs^19–21^. This will allow better understanding of the impact of intervention on the development of protective antibodies.

VSAs such as Plasmodium falciparum erythrocyte membrane protein 1 (PfEMP1) are important targets for opsonising phagocytosis and binding-inhibition^21–23^. Opsonising antibody targets parasite antigens in their natural configuration on the IEs surface, but recombinant VAR2CSA antigens may not be presented in the same way. The declines over pregnancy we observed in levels of VAR2CSA-DBL5 antibodies are similar to those observed with most domains of VAR2CSA in Benin^5^, the exceptions being full-length VAR2CSA and VAR2CSA-DBL6, which both increased over pregnancy. VAR2CSA-DBL5, together with VAR2CSA-DBL2 and VAR2CSA-DBL3, is reported to be an important target of opsonising antibodies^23^, but the differences in dynamics of these responses over pregnancy and responses to intact IEs suggests that IgG assayed by ELISAs may not be a good correlate of more functional antibodies.

When results were stratified according to presence of malaria infection at delivery, women in the IST arm who had malaria infection tended to have higher IgG responses to placental-binding IEs. IST is not presently endorsed by the World Health Organization^24^, in part because current RDTs miss too many low-density infections. Our observations of a modest decrease in IgG antibody to CS2 IEs at delivery in infected women receiving IPT-SP and similar modest increase in opsonising antibody to CS2 IEs in IST-DP recipients are consistent with this, suggesting boosting of antibody responses by infections in women receiving IST but not IPT.

After adjusting for covariates, treatment arms did not influence acquisition and/or maintenance of malarial antibodies over pregnancy. As expected, higher gravidity was associated with increased levels of pregnancy-associated antibodies, indicating more exposure to placental-binding parasites during previous pregnancies. Of note, women who were infected at study entry had higher antibody responses to non-pregnancy related antigens, but had lower antibody responses to placental-binding antigens. An earlier study in Benin demonstrated persistent *P*. *falciparum* infection in nulligravidae, and during the first trimester, these women experienced an increased risk of parasitaemia. Importantly, the administration of IPT decreased the risk of parasitaemia during pregnancy^25^. This possibly suggests infection with endothelial-binding parasites that resulted in boosted antibody responses before placental development, and that anti-malarials administered during second trimester effectively cleared parasitaemia resulting in lower exposure to placental-binding IEs. Of the three study sites, women in Madziabango tended to have the highest malaria antibody responses, consistent with the highest rates of parasitaemia being observed there. Importantly, IST-DP did not modify antibody responses in the different study sites.

Strengths of the study include a large data set of over 600 women whose antibody responses were measured to a range of malaria antibody targets twice in pregnancy. Study limitations include high bed net coverage and frequent IST-DP or IPT-SP, which may have reduced the incidence and density of malaria infection. Second, SP resistance was not determined and parasites were not genotyped, so we are unable to determine whether infection was due to reinfection or recrudescence. Lastly, we did not have blood specimens during ANC visits to assay for the dynamics of antibody responses between each treatment, which could provide further understanding on impact of such interventions.

In this study, we have assessed associations for several antibody responses, resulting in a number of tests and associated p-values. We chose not to penalise our p-values for the multiple testing, as this is a contentious issue in the literature (see for example^26^) but instead we have presented the effect estimate, 95% confidence interval and corresponding p-value for associations of interest and have sought to illustrate comparisons that lead to similar trends of potential relevance. The reader should be cautioned against interpreting these results in terms of statistical significance, and interpret the 95% confidence intervals in terms of the magnitude of the effect and not merely on whether or not they cross the null value^27^.

In conclusion, there was minimal difference in malarial antibody between women receiving IPT-SP and IST-DP at delivery, possibly due to low force of infection and high bed net coverage. The decline between enrolment and delivery in antibody measured against several targets by ELISA supports this. Further studies should explore whether the decline of antibody responses over pregnancy has an impact on the transfer of maternal antibodies to her infant and whether this results in an increase in malaria incidence during infancy. More sensitive diagnostics in the form of regular blood smears and/or better RDTs are required for future evaluation of IST and possibly IPT protocols, as the increases in some antibody responses observed here suggest ongoing malaria exposure, which may lead to poor pregnancy outcomes.

## Materials and Methods

### Ethics approval

The study protocol was reviewed and ethical approval was obtained from Research Ethics Committees of the Liverpool School of Tropical Medicine, the College of Medicine Research Ethics Committee, University of Malawi, and the National Health Sciences Research Committee, Ministry of Health, Malawi. The laboratory studies were approved by the Melbourne Health Human Research Ethics Committee, Australia. The study was conducted in accordance with Good Clinical Practice guidelines (ICH GCP E6) The trial was registered with Pan African Clinical Trials Registry (PACTR201103000280319, 14/03/2011). All participants provided informed written consent^12^.

### Study sites and participants

Full details of the trial were recently published^12^; in brief, this study was conducted in Chikwawa and Blantyre Districts, Malawi. Pregnant women between 16–28 weeks’ gestation and free from chronic illness were randomly assigned to receive IPT-SP or IST-DP, and all were given an insecticide-treated net (ITN). At each of 3 or 4 scheduled antenatal visits, women in the IPT-SP group were given 1,500 mg sulfadoxine and 75 mg pyrimethamine as 3 tablets. Women in the IST-DP group had an RDT performed and if this was positive, received a standard 3-day course of DP by body weight. All treatments in both arms were directly observed. At delivery, placental biopsies were collected, and placental malaria was defined as active infection at delivery by histology. Plasma samples were collected at enrolment and at delivery, and paired samples from 333 participants receiving IPT-SP and 348 receiving IST-DP were assayed for antibody responses against *P*. *falciparum* antigens. All available paired samples were tested.

### Parasites and cell cultures

Placental-binding (CS2) and endothelial-binding (E8B) IEs were cultured as previously described, and were used to measure antibodies to the IEs surface^28,29^.

THP-1 monocyte-like cells were cultured^28^ and used to estimate opsonising antibodies.

### Assay of IgG to recombinant antigens and schizont extract

Plasma samples were assayed by enzyme-linked immunosorbent assay (ELISA) as previously described^30^. In brief, microtitre plates were coated with 50 µL of individual targets diluted in phosphate buffered saline (PBS) at the following concentrations: schizont extract from CS2 IEs 1/2000; MSP2 from FC27 0.5 µg/ml; MSP3 from 3D7 full ectodomain 2 µg/ml; PfRh2 (construct PfRh2-2030 from 3D7) 0.5 µg/ml; and VAR2CSA-DBL5^31^ from 3D7 0.5 µg/ml. Plasma was added in duplicate (1:1000). A standard curve generated from serial dilution of the positive controls (composed of pooled plasma from 44 pregnant women with high antibody responses to CS2 IEs), was used to convert optical density (OD) into antibody level represented in arbitrary units. In each plate, plasmas from six non-exposed pregnant women from Melbourne were included as negative controls.

### Total IgG antibody to variant surface antigens of infected erythrocytes

In brief, test plasma was incubated with placental-binding or endothelial-binding IEs at 4–8% parasitaemia (1:20) in duplicate, followed by incubation with rabbit anti-human IgG (1:100, Dako), and with AlexaFluor 647 donkey anti-rabbit (1:500, Invitrogen) supplemented with 10 µg/ml ethidium bromide (EtBr), in the dark. The cells were re-suspended in 2% paraformaldehyde (PFA) and analysed with flow cytometry^29^. The positive pooled plasma and negative controls used in ELISAs were run on each plate, and antibody to IEs was expressed as relative geometric mean fluorescence intensity (MFI), represented as a percentage of the MFI of the positive pooled plasma, after subtraction of the negative controls.

### Measurement of opsonising antibody against infected erythrocytes

In brief, purified trophozoite-stage IEs stained with 10 µg/ml EtBr were re-suspended at 1.67 × 10^7^/ml and opsonised with test samples (1:10). Opsonised IEs were then aliquoted in duplicates and incubated with THP-1 cells in a 5% CO_2_ humidified incubator at 37 °C. Phagocytosis was arrested and unphagocytosed IEs were lysed before being re-suspended in 2% PFA. Test samples were then analysed with flow cytometry and opsonising antibody was detected as proportion of THP-1 cells positive for EtBr, and expressed as a percentage of positive control^28^.

### Data analysis

Non-parametric continuous variables were analysed using Mann-Whitney U test (Fig. 1) and categorical variables were tested using Chi-squared test (Table 1).

To compare antibody responses at delivery between the two treatment groups (i.e. IST-DP vs IPT-SP), we fitted separate linear regression models for each of the antibody measures. Because the antibody responses were non-normally distributed, the log-transformed antibody responses at delivery were the outcome variables and intervention was the exposure of interest (i.e. IST-DP vs IPT-SP). We assessed if site of intervention at enrolment, gravidity, or presence of *P*. *falciparum* parasitaemia at enrolment modified the association between intervention and each antibody response at delivery by fitting separate interaction terms and testing the interactions with likelihood ratio tests. Our final models included the interaction of intervention (IST-DP vs IPT-SP) with study site, which was found to modify the association between intervention and antibody response (based on a p-value of <0.05) and also adjusted for gravidity, and the presence of *P*. *falciparum* parasitaemia at enrolment (Table 2). To compare antibody responses at enrolment, we fitted linear regression models (as above) but antibody levels at enrolment were now the outcome variable.

To compare change in antibody responses between enrolment and delivery, we fitted a linear regression model. The outcome variable was the change in antibody response between enrolment and delivery in each treatment group (note that this change was determined for women who were infected or uninfected at enrolment or at delivery); treatment (i.e. IST-DP vs IPT-SP) was the exposure of interest and we further adjusted for antibody response at enrolment (Fig. 2; presented overall and by gravidity). To assess how presence of malaria infection at enrolment or delivery modified the change in antibody responses during pregnancy, we presented the results of the quantile regression models by infection status at enrolment (Fig. 3) and at delivery (Fig. 4). To obtain the corresponding standard errors and 95% confidence intervals for the quantile regression models (Figs 2–4), we performed 200 bootstrap replications.

Data were analysed using Stata v14 (StataCorp).

## Supplementary information

Supplementary 1

## Data Availability

The datasets generated and/or analysed in the current study are available from the corresponding author by written request.
