# Supplementary material for: Intermittent screening and treatment with dihydroartemisinin-piperaquine and intermittent preventive therapy with sulfadoxine-pyrimethamine have similar effects on malaria antibody in pregnant Malawian women
Source: Sci Rep. 2019 May 27;9:7878. doi: 10.1038/s41598-019-44340-x (PMC6536723; doi:10.1038/s41598-019-44340-x)
Supplement: Supplementary file 1 — Supplementary 1 [file 41598_2019_44340_MOESM1_ESM.docx]

Intermittent screening and treatment with dihydroartemisinin-piperaquine and intermittent preventive therapy with sulfadoxine-pyrimethamine have similar effects on malaria antibody in pregnant Malawian women

Authors:
Andrew Teo^1,2^*^, Louise M Randall^1,3^, Mwayiwawo Madanitsa^4,5^, Victor Mwapasa^4^, Linda Kalilani Phiri^4^, Carole Khairallah^5^, Christelle Buffet^1,3^, Amalia Karahalios^6^, David L Narum^2^, Feiko O ter Kuile^7^, and Stephen J Rogerson^1,3*^

1. Department of Medicine and Radiology and Doherty Institute, University of Melbourne, Melbourne, Victoria, Australia

2. Laboratory of Malaria Immunology and Vaccinology, National Institute of Allergy and Infectious Diseases, National Institute of Health, Rockville, Maryland, USA

3. Victoria Infectious Diseases Service, Peter Doherty Institute of Infection and Immunity, University of Melbourne, Melbourne, Victoria, Australia

4. College of Medicine, University of Malawi, Blantyre, Malawi

5. Department of Clinical Sciences, Liverpool School of Tropical Medicine, Liverpool, United Kingdom

6. Centre of Epidemiology and Biostatistics, Melbourne School of Population and Global Health, The University of Melbourne, Melbourne, Victoria, Australia

7. Liverpool School of Tropical Medicine, Liverpool, UK

^^^ Former address ^1^, Current address ^2^

^*^ Corresponding author: sroger@unimelb.edu.au and andrew.teo@uqconnect.edu.au

**Keywords:** Plasmodium falciparum, immunity, prevention, Africa, pregnancy, Sulfadoxine-pyrimethamine, dihydroartemisinin-piperaquine

###### Figure S1 Antibodies to P. falciparum antigens in pregnant women at recruitment and delivery***.*** White box –recruitment, grey box –delivery. SP: IPT-SP recipients, N= 333, and DP: IST-DP recipients, N=348. (**A)** Levels of IgG to schizont extract, MSP2, MSP3, PfRH2 and 3D7-DBL5. **(B)** Total levels of IgG to variant surface antigens of endothelial-binding and placental-binding infected erythrocytes (IEs). **(C)** Levels of opsonising IgG to variant surface antigens of endothelial-binding and placental-binding IEs, presented as percentage of THP-1 cells that have ingested IEs (percentage of phagocytosis). Wilcoxon signed-rank test, **** p<0.0001, *** p<0.001 and * p<0.05. Box and line show median and IQR, and whiskers 10 – 90 percentiles with outliers in closed circles
